# Discovery of a new ant species of the elusive termitophilous genus *Metapone* in Singapore (Hymenoptera, Formicidae, Myrmicinae), with the first detailed description of male genitalia of the genus

**DOI:** 10.3897/zookeys.876.35739

**Published:** 2019-09-25

**Authors:** Wendy Y. Wang, Aiki Yamada, Katsuyuki Eguchi

**Affiliations:** 1 Lee Kong Chian Natural History Museum, National University of Singapore, 2 Conservatory Drive, 117377, Singapore National University of Singapore Singapore Singapore; 2 Systematic Zoology Laboratory, Department of Biological Sciences, Graduate School of Science, Tokyo Metropolitan University, 1-1 Minami-Osawa, Hachioji-shi, Tokyo, 192-0397, Japan Tokyo Metropolitan University Tokyo Japan

**Keywords:** Museum specimens, rare genus, taxonomy, nest series

## Abstract

A new species of the rare ant genus *Metapone*, *Metapone murphyi***sp. nov.**, is described based on museum material consisting of a single nest series (workers, queens, and males) collected from a decayed coconut palm stump on Pulau Sakra, previously an offshore island south of mainland Singapore. Workers can be distinguished from other named congeners mainly by the following characters: 1) subpetiolar lamella subrectangular; 2) short median longitudinal ventral subpetiolar edge and roundly obtuse posteroventral subpetiolar angle; 3) outer margin of posterior subpetiolar face in posteroventral view forming a continuous, U-shaped, translucent, laminate carina; and 4) petiole subtrapezoidal in dorsal view with extended blunt tooth-like posterolateral corners. Detailed description and illustrations of male genitalia of the genus are given for the first time. The key to Asian species of *Metapone* is updated to include the new species.

## Introduction

The myrmicine genus *Metapone* Forel, 1911 is known from tropical Africa, Madagascar, Sri Lanka, the Philippines, archipelagic Southeast Asia, Melanesia east to Fiji and Eastern Australia (Guénard et al. 2017). From Asia and Southeast Asia, nine nominal species have been reported: *M. bakeri* Wheeler, 1916 (Philippines, Luzon), *M. balinensis* Taylor & Alpert, 2016 (Indonesia, Bali), *M. greeni* Forel, 1912 (Sri Lanka), *M. hewitti* Wheeler, 1919 (Malaysia, Sarawak), *M. jacobsoni* Crawley, 1924 (Indonesia, Sumatra), *M. javana* Taylor & Alpert, 2016 (Indonesia, Java), *M. quadridentata* Eguchi, 1998 (Malaysia, Sabah), *M. sauteri* Forel, 1912 (Taiwan), and *M. wallaceana* Taylor & Alpert, 2016 (Indonesia, Lombok). There are no past records from continental Southeast Asia (Taylor and Alpert 2016). This genus is rarely collected by myrmecologists, and generally presumed to be termitophilous, since colonies are usually encountered in close association with termite nests (Taylor and Alpert 2016).

We herein describe a new species, *Metapone murphyi* sp. nov., from Singapore, based on a nest series which was found stored in ethanol as part of the Zoological Reference Collections (ZRC), in the Lee Kong Chian Natural History Museum (National University of Singapore). Multiple queens (five of eight), two workers (two of three; one headless), and two males were dry mounted for morphological examination. The present paper provides the detailed description of the male genitalia of *Metapone* for the first time: this information will be valuable for future comparative studies on this understudied genus. In addition, the existing key to Asian species of *Metapone* (Taylor and Alpert 2016) is also updated to include the new species.

## Materials and methods

Morphological observations were made using an Olympus SZX16 stereomicroscope, while measurements were made using micrometres (ocular graticule) on the same microscope. Genitalia of one male were slide-mounted according to the preparation steps described in Yamada and Eguchi (2016), and examined with a Nikon Eclipse E600 microscope.

### Measurements and indices

Measurements in mm, mostly adapted from dimensions in Taylor and Alpert (2016):

**TL** Total aggregate outstretched length of the ant from mandibular apex to gastral apex in lateral view.

**HL** Maximum length of head capsule excluding the mandibles, measured in full-face view in a straight line from the mid-point of the occipital margin to the limits of clypeal projection.

**HW** Maximum width of head in full-face view, across eyes when applicable.

**CpL** Maximum clypeal length as measured from the anterior to posterior clypeal margins.

**EL** Maximum eye length measured across its maximum diameter in lateral view.

**MSL** Length of mesosoma in lateral view, measured as a straight line approximately parallel to the dorsal outline of the mesosoma, from the furthest anterodorsal point of the pronotum (including anterior slope) to the posterior limit of the propodeum.

**PML** Maximum length of promesonotal disc in dorsal view, measured along the midline originating from anterior margin of promesonotal disc (excluding anterior slope of promesonotum).

**PMW** Maximum width of promesonotal disc in dorsal view.

**PDW** Maximum width of propodeum in dorsal view.

**PetL** Maximum length of petiolar node in dorsal view, including posterolateral extensions if present.

**PetW** Maximum width of petiolar node in dorsal view, including posterolateral extensions if present.

**PetH** Maximum height of petiolar node, measured as a direct straight line connecting its dorsal and ventral extremities in lateral view, including the subpetiolar extension.

**PpetL** Maximum length of postpetiolar dorsum (excluding helcium) in lateral view, including antero- and/or posterolateral extensions of the postpetiolar disc if present.

**PPetW** Maximum width of postpetiolar dorsum, measured in dorsal view.

**PPetH** Maximum height of postpetiole, measured as a direct straight line connecting its dorsal and ventral extremities.

**GW** Maximum width of first gastral segment, measured in dorsal view.

**CI** Cephalic Index, HW/HL × 100.

**CpI** Clypeal Index, CpL/HL × 100.

**PMI** Promesonotal Index, PML/MSL × 100.

**REL** Relative Eye Length, EL/HW × 100.

Additional measurements applicable to gyne mesosoma:

**PnL** Length of pronotal disc, measured along the midline originating from anterior margin of promesonotal disc (excluding anterior slope of pronotum).

**PnW** Maximum width of pronotal disc, measured across the widest part of the disc in dorsal view.

**ScL** Length of mesoscutum, measured along the midline of the scutum in dorsal view.

**ScW** Width of mesoscutum, measured across the widest part of the sclerite in dorsal view.

Specimen depositories, collections, and their abbreviations:

**MNHAH**Museum of Nature and Human Activities, Hyogo, Japan.

**SKYC** Seiki Yamane Collection, Japan.

**ZRC**Zoological Reference Collection, Lee Kong Chian Natural History Museum, Singapore.

### Additional *Metapone* material examined

**Types.***Metapone quadridentata* Eguchi, 1998. Three paratypes from East Malaysia (Borneo), Sabah, Poring, Kinabalu were examined. Two paratype workers (SKYC) and one paratype queen (MNHAH).

Source images of the new species for focus stacking were taking using a Canon EOS Kiss X9 digital camera, attached to a Nikon AZ100 stereomicroscope (for worker, queen, and male bodies, excluding male genitalia), and a Nikon Eclipse E600 microscope (for male genitalia). Focus-stacked images were produced using Helicon Focus Pro 7.0.2 (Helicon Soft Ltd., http://www.heliconsoft.com/), and improved with the retouching function of the same software. Colour balance and contrast were adjusted using GIMP 2.8 (The GIMP Development Team, http://www.gimp.org). Two paratype workers of *M. quadridentata* were imaged on a Dun Inc™ Passport II macrophotography imaging system, using a Canon MP-E 65 mm lens. Focus-stacked images of the two paratypes were produced using Zerene Stacker (Zerene Systems LLC, http://zerenesystems.com/cms/stacker). The final images were further adjusted, annotated, and scale bars added using Adobe Photoshop CS6. All specimens of the new species examined are deposited in the Lee Kong Chian Natural History Museum, under the Zoological Reference Collection (ZRC).

High resolution image plates are available at https://doi.org/10.5061/dryad.9776725

## Taxonomic accounts

### Metapone murphyi sp. nov

Taxon classificationAnimaliaHymenopteraFormicidae

C033C0EE-1BB7-5A4A-B8C3-CFB9B43F125B

http://zoobank.org/6FC954CB-057C-4430-8F32-63825B750A64

**Figures 1–4.**
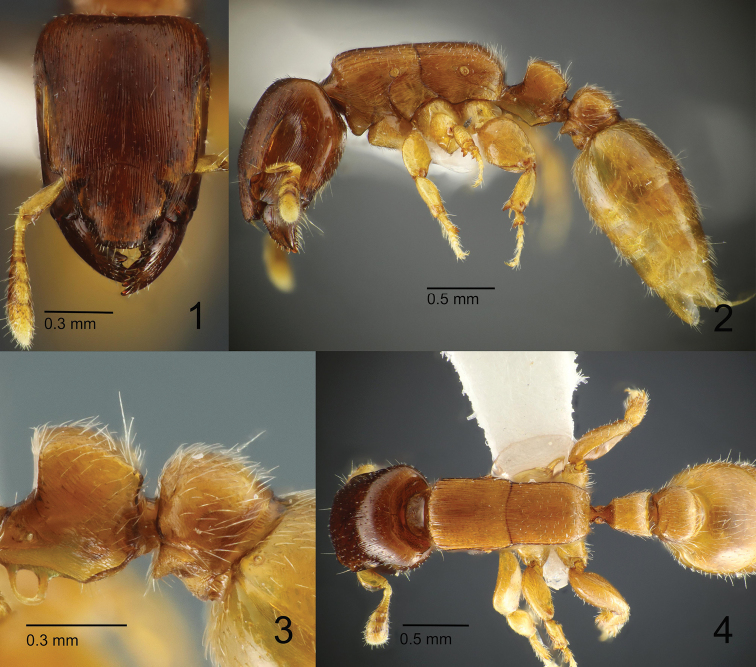
*Metapone murphyi*, holotype **1** head in full-face view **2** body in lateral view **3** waist segments in lateral view **4** mesosoma and waist in dorsal view.

Figs 1 , , 9–12 , 13–19 , 20–23

#### Types.

***Holotype:*** Worker. SINGAPORE, Pulau Sakra (1.2592°N, 103.7042°E), decayed coconut stump, 7.March.1981, D.H. Murphy leg., colony code: DHM-81-Metapone, depository catalogue number: ZRC_ENT_00000878 (ZRC). ***Paratypes***: Two workers (1 headless), 8 alate queens, 2 males, same colony as the holotype, depository catalogue number: ZRC_HYM_0000016.01–11 (ZRC).

#### Diagnosis.

***Worker.*** (1) Body, small-sized, monomorphic with broad size variation (HL 0.98–1.24, HW 0.75–0.93). (2) Head subrectangular, with lateral sides nearly entirely straight and parallel; (3) anterior margin of rostrate projection of clypeus faintly crenate; (4) petiolar node in lateral view subrectangular, in dorsal view subtrapezoidal, slightly wider than long, with posterolateral corners extended to form tooth-like projections; (5) subpetiolar lamella small and subrectangular, dorsoventrally slightly higher than anteroposteriorly wide, thin and translucent; (6) short median longitudinal ventral subpetiolar edge meeting the broadly rounded lateral outline of posterior subpetiolar face at a roundly obtuse posteroventral subpetiolar angle; (7) outer margin of posterior subpetiolar face forming a continuous, U-shaped, translucent, laminate carina in posteroventral view. (8) Head and mesosoma mostly densely striated longitudinally and shining.

***Queen.*** (1) Body relatively small-sized (HL 1.28–1.33, HW 0.85–0.93); (2) posterolateral corners of head faintly striated and shining; (3) petiolar node subrectangular in lateral view, elongate-subtrapezoidal in dorsal view, more than twice as long as its anterior margin; (4) lateral face of petiolar node substriate-reticulate with few long diagonally-placed carinae; (5) subpetiolar lamella, subpetiolar edge and posterior subpetiolar face as in the worker.

**Worker measurements.** Holotype worker (small worker): TL 4.60, HL 0.98, HW 0.75, CpL 0.38, EL 0.10, MSL 1.18, PML 0.60, PMW 0.55, PDW 0.45, PetL 0.30, PetW 0.35, PetH 0.50, PpetL 0.33, PpetW 0.40, PpetH 0.43, GW 0.75, CI 77, CpI 38, PMI 51, REL 13.

Paratype worker (large worker; ZRC_HYM_0000016.01), body from petiole to gaster missing: HL 1.24, HW, 0.93, CpL 0.49, EL 0.18, MSL 1.60, PML 0.85, PMW 0.70, PDW 0.55, CI 75, CpI 40, PMI 53, REL 19.

**Queen measurements.** Four paratype queens (n = 4): TL 6.4–6.9, HL 1.28–1.33, HW 0.85–0.93, CpL 0.5–0.53, EL 0.38–0.40, MSL 1.90–2.05, PML 1.30–1.40, PMW 0.75–0.88, PDW 0.50–0.55, PetL 0.48–0.55, PetW 0.40, PetH 0.65–0.75, PpetL 0.40, PpetW 0.58–0.63, PpetH 0.53–0.58, GW 0.80–0.90, CI 67–70, CpI 38–40, PMI 65–68, REL 43–44, PnL 0.65–0.70, PnW 0.70–0.83, ScL 0.60–0.68, ScW 0.75–0.80.

**Male measurements.** One paratype male (n = 1): TL 4.5, HL 0.90, HW 0.93, CpL 0.40, EL 0.43, MSL 1.60, PML 1.20, PMW 0.80, PDW 0.50, PetL 0.25, PetW 0.40, PetH 0.58, PpetL 0.33, PpetW 0.50, PpetH 0.43, GW 0.85, CI 103, CpI 44, PMI 75, REL 46, PnL 0.60, PnW 0.75, ScL 0.75, ScW 0.75.

**Figures 5–8.**
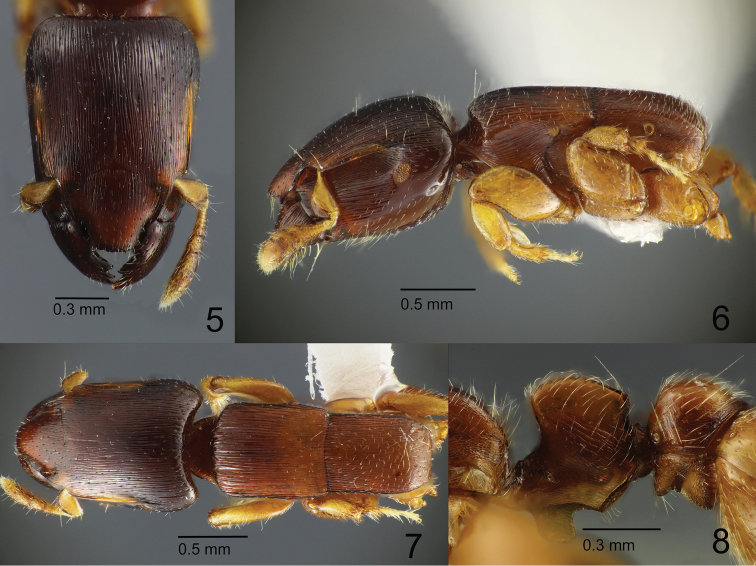
*Metapone murphyi***5–7** large paratype worker (ZRC_HYM_0000016.01) **5** head in full-face view **6** body in lateral view (waist segments and gaster missing) **7** mesosoma in dorsal view **8** paratype worker, headless (ZRC_HYM_0000016.02) waist segments in lateral view.

#### Description.

***Worker.*** Body small-sized (HL 0.98–1.24, HW 0.75–0.93), monomorphic with distinct variation in size. Head in full-face view subrectangular; posterior margin very weakly concave; posterolateral corners round; lateral margins almost entirely straight. Eye elongate-elliptical, relatively small, larger in large worker in proportion to head length, positioned far behind the midlength of head, ventrad to antennal scrobe, close to the level of the posterior end of antennal scrobe; REL 13 in holotype, 19 in a paratype (large worker). Mandible large and dorsoventrally high in lateral view; masticatory margin with four robust teeth roughly uniform in size; basal angle well-developed. Clypeus large; median portion of clypeus extended forward, forming a short rostrate projection; the projection slightly narrowed anteriorly, with anterior margin faintly convex, lateral margin broadly concave, and anterolateral angles subdentate. Frontal carinae parallel, well developed, partly overhanging antennal scrobe which accommodates antennal scape. Antenna 11-merous; scape short, roughly reaching the midlength of head when laid backward, flattened and dilated apically, with anterior part of leading edge forming a translucent lamella; funiculus flattened; apical 3 antennomeres forming a club. Mesosoma elongate rectangular in lateral view, with dorsal outline almost entirely straight. Promesonotal suture absent; anterior margin of promesonotal disc in dorsal view broadly and roundly convex, in lateral view extended to form a short overhang above pronotal neck; lateral margins of promesonotal disc in dorsal view almost entirely straight and parallel; humeral corner roundly angulate. Mesothoracic spiracle in lateral view almost spherical, in dorsal view slightly protruding from the lateral outline of mesosoma. Metanotal groove present as a thin but distinct sulcus which is slightly convex anteriorly in dorsal view. Propodeum in dorsal view a little narrower than promesonotum; lateral margins almost parallel in the anterior half and then converging posterad; posterior margin almost straight; propodeal junction in lateral view with angle round and obtuse. Petiolar node in lateral view subrectangular, longer than high, in dorsal view subtrapezoidal, almost as long as wide, with anterior margin straight or weakly concave, posterior margin weakly concave, and lateral margins almost straight and diverging posterad; anterolateral corner in dorsal view obtusely angulate; posterolateral corner in dorsal view angulate and weakly produced posterad; in posterior view lateral margins slightly converging from dorsum to base without distinctly curving inwards near the dorsum. Subpetiolar lamella subrectangular, thin and translucent in a paratype (large worker; Fig. 8), or with a large central opening in the holotype (Fig. 3), with anteroventral corner broadly round, with posteroventral corner narrowly round and slightly produced posteroventrad; the ventral subpetiolar edge in lateral view short, forming an obtuse, rounded angle (posteroventral subpetiolar angle) with outer margin of posterior subpetiolar face; outer margin of posterior subpetiolar face in posteroventral view forming a continuous, U-shaped, translucent and laminate carina. Postpetiole in dorsal view globular, broader than long, slightly broader than petiole, with anterior margin weakly concave; sub-postpetiolar process in lateral view moderately and triangularly produced. Legs short and stout; femora flattened anteroposteriorly, high dorsoventrally; anteroventral part forming a groove which is margined with carinae and partly accommodates tibia; protibia with a tuft of short hairs anterior to large pectinate spur and a short spine behind the spur; meso- and metatibia each with a much smaller pectinate spur and two large spinose setae anterior to the spur; basitarsus stout; probasitarsus posteroapically with three large spinose setae and one small spinose seta; meso- and metabasitarsus anteroapically with four large spinose setae.

**Figures 9–12.**
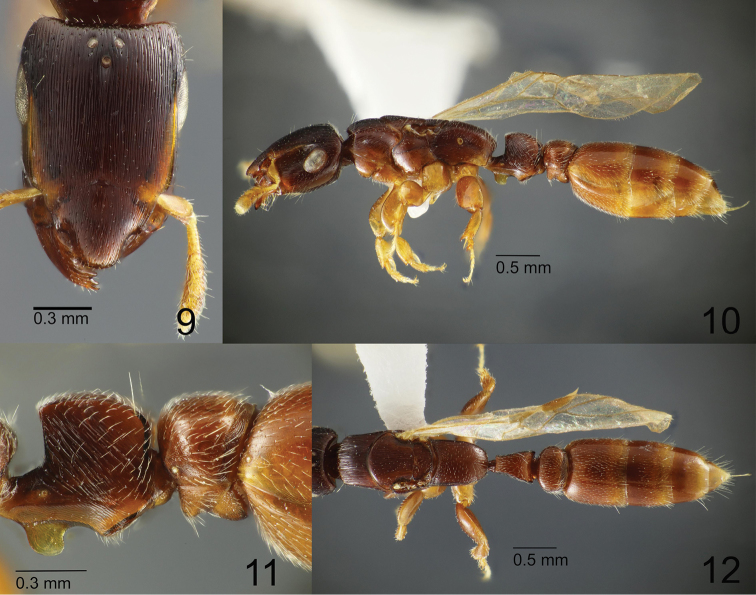
*Metapone murphyi*, paratype alate queen (ZRC_HYM_0000016.03) **9** Head in full-face view **10** body in lateral view **11** waist segments in lateral view **12** mesosoma and waist in dorsal view.

Dorsum and anterior half of lateral face of head, and antennal scrobe densely striate and shining; mandible striate-punctate and shining; posterolateral corners and ventral face of head mostly smooth and shining. Dorsum and lateral face of mesosoma densely striated and largely shining; interspaces in metapleuron and lateral face of propodeum faintly punctate. Petiole with lateral face weakly striate-punctate and shiny, and dorsal face largely smooth and shining. Postpetiole with lateral face shagreened, and dorsal face largely smooth and shining. Gaster largely smooth and shining; anterior part of tergites II–IV faintly and transversely imbricate.

Dorsum of head bearing very short, sparse, decumbent hairs, with a few longer erect hairs; ventral and lateral faces of head with numerous short decumbent hairs; posterior border of hypostoma and ventral margin of mandible with linearly-arranged long bristle-like setae. Rostrate projection of clypeus with several long bristle-like setae beneath the anterior margin. Dorsum of mesosoma bearing very short, sparse, decumbent hairs, with a few longer erect hairs; lateral face of mesosoma almost hairless; propodeal declivity with many long erect hairs. Petiolar node with dense and long decumbent hairs on upper part of anterior face, with sparse standing hairs on dorsal face; each postero-dorsolateral corner with a long erect hair. Postpetiole and gaster with abundant standing and subdecumbent hairs of roughly uniform length, with sparse longer erect hairs.

Entire body generally brown in colour; head darker than remainder of body; antenna and legs more yellowish; tip and joint of antenna paler than remainder of antenna. Whole body darker and more reddish in large worker than in small worker.

***Queen.*** Body similar in size to the large worker (HL 1.28–1.33, HW 0.85–0.93). Head in full-face view subrectangular, more elongate than in the worker (CI 67–70 in the queen, 75–77 in the worker); posterior margin weakly and broadly concave; posterolateral corners rounded, slightly more angulate than in the worker; lateral margins almost entirely straight and parallel. Eye very large and elongate, in full-face view located behind the midline of head, very weakly convex and protruding from the lateral outline of head. Ocelli very small; median ocellus located around the level of the posterior ends of frontal carinae. Mandible as in the worker. Clypeus as in the worker; rostrate projection a little more slender in the queen than in the worker. Frontal carina, antennal scrobe, and antenna as in the worker. Mesosoma elongate rectangular, more slender than in the worker, in lateral view with dorsal outline weakly and broadly convex. Anterior margin of pronotal disc in dorsal view broadly and roundly convex, in lateral view extended to form a short overhang above pronotal neck; lateral margins of the disc almost straight or slightly concave, weakly diverging posterad; humeral corner roundly angulate. Mesoscutum wider than long, slightly longer than pronotal disc; notaulus absent; parapsidal signum weakly present; transscutal articulation recognized as a transverse weakly curved scutoscutellar sulcus spanning almost the entire posterior mesoscutal margin; axilla distinctly separated from mesoscutellum by a curved sulcus; posterior margin of mesoscutellum roundly convex. Mesopleuron distinctly divided into upper and lower part by a weakly sinuate sulcus. Propodeum in dorsal view longer than wide, with anterior margin broadly and roundly concave, with lateral margins almost parallel in the anterior 3/5 and then converging posterad, with posterior margin roundly convex; dorsal outline of propodeal dorsum in lateral view entirely downward-sloping; propodeal junction in lateral view roundly and weakly produced over posterior propodeal face. Petiolar node in lateral view subrectangular, longer than high, in dorsal view elongate subtrapezoidal, less than twice as long as wide, with anterior margin straight or weakly concave, posterior margin weakly concave, and lateral margins slightly sinuate and diverging posterad; anterolateral corner in dorsal view almost right-angled; posterolateral corner in dorsal view angulate and weakly produced posterad; subpetiolar lamella, subpetiolar edge and posterior subpetiolar face as in the worker. Postpetiole as in the worker; subpostpetiolar process in lateral view weakly and bluntly extending anteroventrad. Legs as in the worker.

Sculpture, pilosity, and body colour as in the worker.

**Figures 13–19.**
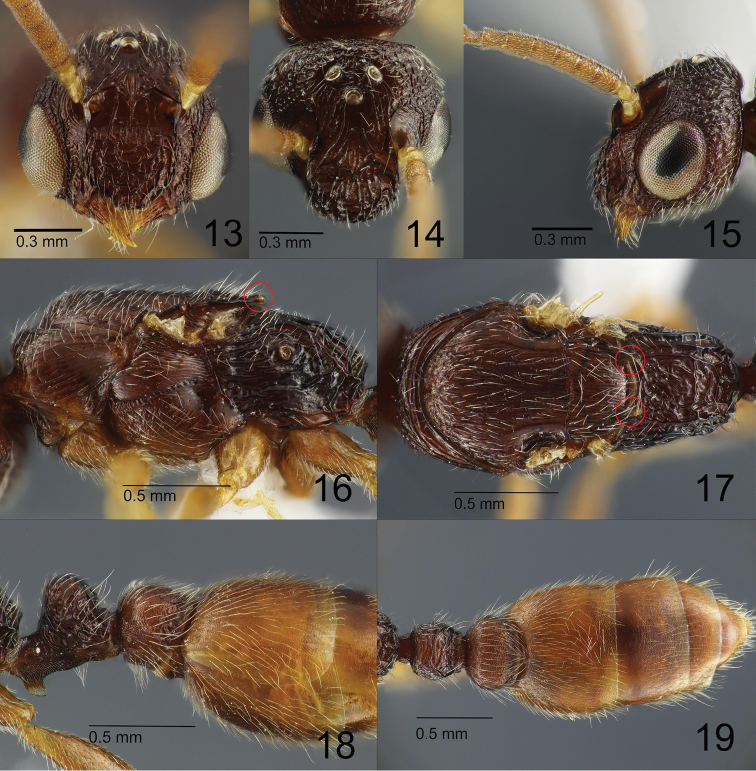
*Metapone murphyi*, paratype male (ZRC_HYM_0000016.10) **13** head in full-face view **14** head in subdorsal view **15** head in lateral view **16** mesosoma in lateral view **17** mesosoma in dorsal view **18** metasoma in lateral view **19** metasoma in dorsal view. Posterior tooth-like extensions of the mesoscutellar disc are encircled in red.

***Male.*** Body small-sized (HL 0.90, HW 0.93). Head in full-face view (Fig. 13) subovate, with posterior margin very roundly convex, in anterodorsal view (Fig. 14) subtriangular, with posterolateral corners and anterior margin broadly round, in lateral view relatively high dorsoventrally (Fig. 15). Occipital carina distinct laterally, less distinct dorsally. Eye very large, in full-face view with posteriormost end reaching or slightly surpassing the midlength of head; in full-face view lateral outline of eye evenly convex and protruding from the lateral outline of head. Ocelli developed more than in the queen; median ocellus located around the level of the posterior ends of frontal carinae (Fig. 14); the distance between the median and lateral ocelli a little shorter than that between lateral ocelli. Mandible with distinct basal and masticatory margins; masticatory margin 3-toothed, with a large apical tooth followed by slightly smaller preapical tooth and minute 3^rd^ tooth. Anterior clypeal margin as a whole roughly M-shaped (Fig. 13); median part of clypeus extended forward to form a short rostrate process; rostrate process narrowed anteriorly, partly overhanging mandible, with an almost straight anterior margin, and lateral margin carinate and weakly sinuate; clypeus in lateral view broadly and evenly convex anterodorsad. Frontal carina short and lamellate; antennal scrobe short but deep. Antenna 12-merous; scape very short and small; 2^nd^ and 3^rd^ antennomeres greatly reduced and extremely small; 3^rd^ antennomere shorter than 2^nd^. Mesosoma in dorsal view elongate sub-elliptical, widest at the level of posteriormost ends of pronotum, in lateral view elongate-rectangular. Anterior margin of pronotal disc in dorsal view broadly and roundly convex, extended to form a short overhang above pronotal neck; lateral margins of the disc in dorsal view broadly and roundly convex; humeral corner present but indistinct. Mesoscutum in dorsal view a little wider than long, with anterior margin strongly and roundly convex; notaulus and parapsidal signum absent; transscutal articulation recognized as a transverse weakly curved scutoscutellar sulcus spanning almost the entire posterior mesoscutal margin; axilla distinctly separated from mesoscutellum by a curved sulcus; posterior margin of mesoscutellar disc with a pair of posteriorly-pointing short tooth-like extensions. Mesopleuron distinctly divided into upper and lower parts by a weakly sinuate sulcus. Propodeum in dorsal view longer than wide, with anterior margin broadly and roundly concave; lateral margins almost straight, converging posterad; posterior margin roundly convex; dorsal outline of propodeal dorsum roundly down-sloping; propodeal junction in lateral view angulate. Petiolar node in lateral view slightly higher than long, with parabolic dorsal outline, in dorsal view sub-oval, broader than long, with weakly concave anterior and posterior margins; subpetiolar lamella small, semi-translucent and subrectangular, a little higher than long, with round anteroventral corner and strongly produced posteroventral corner; ventral subpetiolar edge and posteroventral subpetiolar angle reduced; posterior subpetiolar face in posteroventral view recognized as an isosceles triangle. Postpetiole in dorsal view subelliptical, nearly twice as wide as long, slightly longer than and wider than petiolar node, with roundly convex lateral margins. Gaster in dorsal view sub-elliptical, tapering apicad. Legs moderately long; pro- and meso- femora distinctly longer than pro- and meso-tibiae respectively, but metatibiae almost as long as metafemora; femora each with weak apicoventral lamellate flange; protibiae each with one large pectinate ventroapical spur; mid- and metatibiae each with one simple ventroapical spur; pretarsal claw simple and strongly curved, such that its apex is directed almost perpendicularly upwards from its base; arolium small.

Pygostyle digitiform, with 4–5 long hairs on its apex. Abdominal sternite IX (subgenital plate) 1.7 times as long as wide (including spiculum); posterior disc subparabolic, with posterolateral corner weakly produced; outer face of apical part of disc with several hairs; spiculum long, 0.4 times as long as entire length of the sternite IX (spiculum length measured from the apex to the transverse line spanning the posteriormost points of each side of the anterolateral margin of the posterior disc). Cupula well-developed, 1.6 times as wide as long in dorsal view. Telomere in lateral view subparabolic, distinctly longer than high, without clear articulation to basimere. Gonostipital arm well-developed, with acute apex; lower basimere with conspicuous oblique carina (BmC in Fig. 22). Cuspis well developed, forming subtriangular lobe in lateral view with 2 hairs near apex. Digitus in lateral view elongate-digitiform, entirely hooked ventrad, with blunt apex; mesal face of the apical part with very short hairs (sockets are recognized in Fig. 22); ventral margin of volsella with 12–14 long hairs. Valviceps in lateral view a little longer than wide, gently tapering posterad, forming a broadly rounded apex; anteroventral corner weakly produced; ventral margin serrated with 15 denticles.

Head mostly rugoso-reticulate, with interspaces smooth and shining; dorsal face of rostrate projection rugoso-areolate, with interspaces microsculptured and shining. Mandible carinate, with punctate and shiny interspaces. Dorsum of pronotum and anterior border of mesoscutum irregularly reticulate; the remainder of mesoscutum, mesoscutellum and upper part of mesopleuron longitudinally rugoso-reticulate; lateral face of pronotum longitudinally rugose. Propodeum coarsely rugoso-reticulate; interspaces with sparse punctures and shining. Anterior face of petiole and subpetiolar process finely microreticulate and weakly shining; dorsal and lateral faces of petiolar node rugoso-reticulate, with interspaces microsculptured and shining. Postpetiole and gastral tergites I–III (excluding anterior borders) finely microreticulate and nearly matte.

Head, dorsal and lateral faces of mesosoma, petiole, postpetiole, and gaster covered by short standing hairs (Figs 13–19).

Body except gaster deep reddish brown; gaster lighter orange-brown. Mandible mostly light brown, becoming yellowish toward masticatory margin. Legs and antennae light yellowish-brown, becoming paler toward the apex.

**Figures 20–23.**
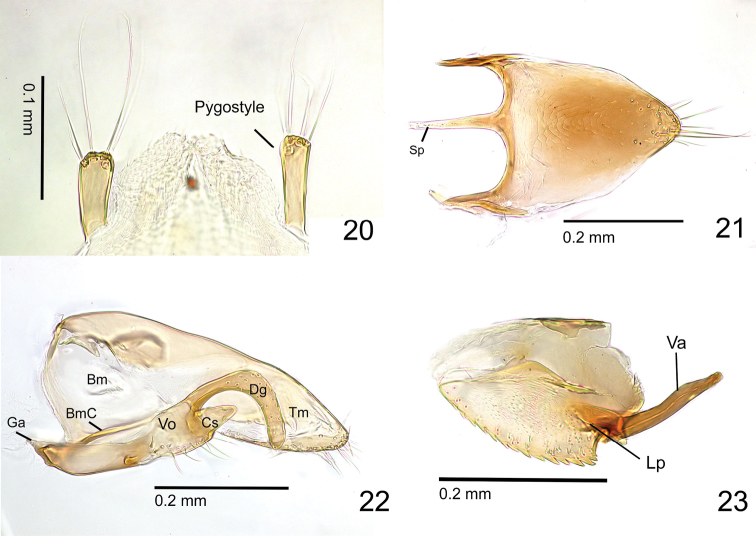
Male genitalia of *Metapone murphyi*, paratype male (ZRC_HYM_0000016.11) **20** Pygostyle in dorsal view **21** abdominal sternite IX in ventral view **22** paramere and volsella, right side, inner view **23** penisvalva, right side, in outer view. Abbreviations: Bm basimere; BmC carina of lower basimere; Cs cuspis; Dg digitus; Ga gonostipital arm; Lp lateral apodeme of penisvalva; Sp spiculum; Tm telomere; Va valvura; Vo volsella.

#### Etymology.

The specific name is dedicated to Prof. D.H. (‘Paddy’) Murphy, who collected a huge number of ants (including the type series of *M. murphyi*) and other insects in Singapore, many of which make up a substantial and invaluable part of the ZRC, collectively referred to informally as ‘the Murphy Collection’.

#### Habitat.

The offshore island where *M. murphyi* was collected, Pulau Sakra, used to be mostly forest and swampland, before being merged with other islands in close vicinity and developed for establishment of oil refining facilities after 1985.

#### Remarks.

The worker of *M. murphyi* (hereafter referred to as MM-w) is most similar in morphology to that of *M. quadridentata* Eguchi, 1998 (MQ-w) (Figs 24–31), but is distinguishable from the latter by the following features:

1) Slope of propodeal declivity in dorsal view weakly concave and depressed medially in MQ-w, but weakly convex and almost entirely flat in MM-w.

2) Petiolar node in posterior view with lateral margins sharply curving inwards from the dorsum of the node then downwards to the base of the node in MQ-w, but slightly converging from the dorsum to the base without distinctly curving inwards in MM-w.

3) Posteroventral corner of subpetiolar lamella strongly produced posteroventrad in MQ-w (Figs 26, 30), but only slightly in MM-w (Figs 3, 8).

4) Mesothoracic spiracle of large worker more elongate-oval in MQ-w (Fig. 25), but more spherical in MM-w (Fig. 6).

5) Propodeal lobes present as low but distinct and near-translucent lamellate flanges in MQ-w, but absent in MM-w.

The queen of *M. murphyi* (hereafter referred to as MM-q) is also most similar in morphology to that of *M. quadridentata* Eguchi, 1998 (MQ-q), but distinguishable from the latter by the following features:

1) MQ-q is distinctly larger in size than MM-q (MQ-q: TL 10.1, HL 1.91, MSL 3.04; MM-q: TL 6.4–6.9, HL 1.28–1.33, MSL 1.90–2.05). Body also stouter in MQ-q than in MM-q.

2) Posterior corner of propodeum in dorsal view forming a smoothly round angle which clearly separates lateral and posterior margins of propodeum in MQ-q, but not forming a distinct angle in MM-q.

3) Anterior margin of petiolar node in dorsal view weakly convex, with rounded anterolateral corner in MQ-q, but straight or weakly concave, with angulate corner in MM-q.

**Figures 24–27.**
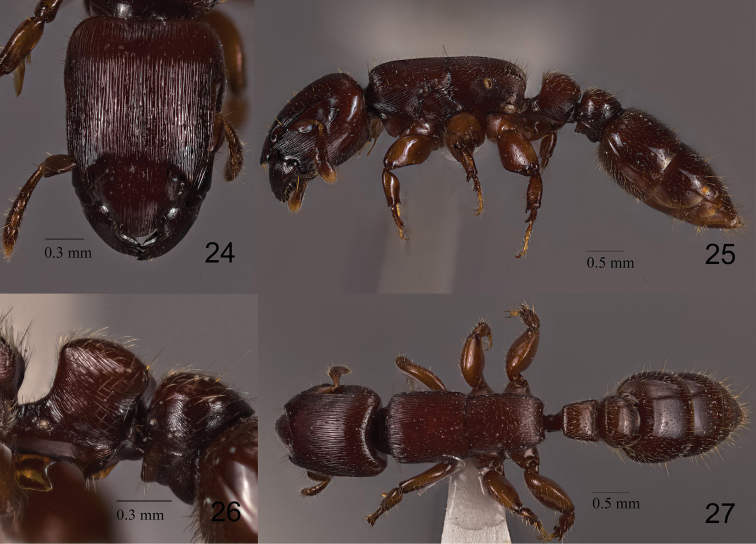
*Metapone quadridentata*, paratype, large worker **24** head in full-face view **25** body in lateral view **26** waist segments in lateral view **27** body in dorsal view.

4) Subpetiolar lamella in lateral view distinctly longer than high, with anteroventral and posteroventral corners roundly angulate and separating anterior, ventral and posterior margins in MQ-q, but slightly longer than high, with anterior, ventral and posterior margins forming a continuous rounded outline in MM-q.

5) In MQ-q, posteroventral subpetiolar angle (including outer margin of posterior subpetiolar face) in lateral view with the apex slightly extended as a sharply-pointed process; in MM-q, posteroventral subpetiolar angle obtuse and not pointed.

6) Dorsal outline of propodeum largely flat, with posterior half slightly down-sloping in MQ-q, but broadly convex, and evenly, though weakly, down-sloping in MM-q.

7) PpetL/PetL = 0.90 in MQ-q, but 0.73–0.84 in MM-q.

8) Dorsum of pronotal neck with dense hairs in MQ-q, but with sparse or almost no hairs in MM-q.

9) Anterior margin of pronotal disc in lateral view not extended over the pronotal neck in MQ-q, but extended slightly over the pronotal neck forming a short overhang in MM-q.

The male of *M. murphyi* (MM-m) is distinguishable from that of the known male-based species, *M. hewitti* (MH-m), by the following characters; see also Wheeler (1919) and Taylor and Alpert (2016):

1) Masticatory margin of mandible with 4 subequal teeth in MH-m, but with 3 teeth of different sizes in MM-m.

2) 2^nd^ antennomere (pedicel) distinctly broader than long in MH-m, but globular and almost as broad as long in MM-m.

3) Petiolar node in lateral view cuboidal, with dorsal outline nearly flat in MH-m, but with parabolic dorsal outline in MM-m.

4) Subpetiolar lamella subtriangular in MH-m, but subrectangular, with round anteroventral corner and strongly produced posteroventral corner in MM-m.

5) Posteroventral subpetiolar angle in lateral view obtuse, with a low apex in MH-m, but reduced and indistinct in MM-m.

**Figures 28–31.**
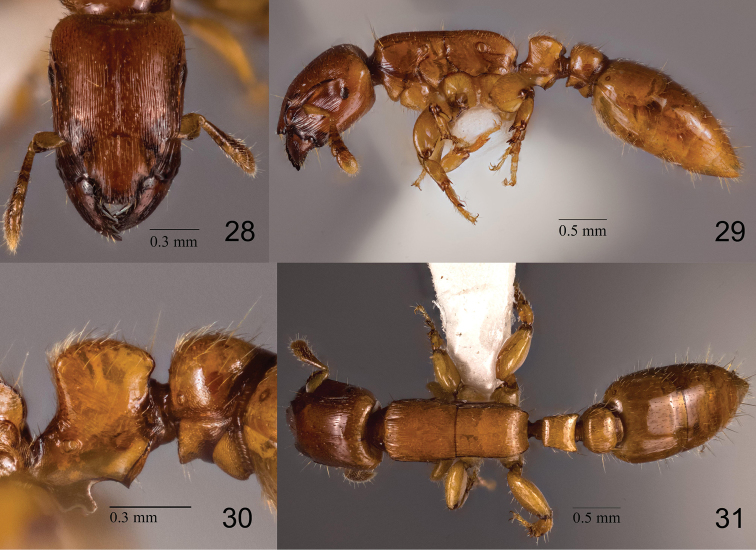
*Metapone quadridentata*, paratype, small worker **28** head in full-face view **29** body in lateral view **30** waist segments in lateral view **31** body in dorsal view.

### Modification to the key to the Asian species of *Metapone*

The key to the Asian species of *Metapone* given by Taylor and Alpert (2016) is herein partly modified below; as a result of these modifications the couplet “8(2)” in the original key becomes couplet “9(2)”.

**Table:** 

5	Posterior face of subpetiolar process relatively large, with a distinct translucent framing lamella in lateral view	**6**
–	Posterior face of subpetiolar process reduced and relatively small; in lateral view posteroventral subpetiolar angle either obtuse, or indistinct with the reduced posterior face transiting directly into the broadly-rounded profile of the ventral subpetiolar edge	**8**
6	Posteroventral subpetiolar angle in lateral view distinct, slightly extended as a pointed process	**7**
–	Ventral subpetiolar edge in lateral view forming an obtuse, rounded angle (posteroventral subpetiolar angle) with outer margin of posterior subpetiolar face	***M. murphyi* sp. nov**.
7	Anteroventral subpetiolar extension relatively large, longitudinally subrectangular with slightly rounded corners; outline of posterior subpetiolar face in lateral view steeply sloping and broadly concave, curved downwards to meet apex of posteroventral subpetiolar angle (Sri Lanka, gyne and worker)	***M. greeni* Forel**
–	Anteroventral subpetiolar extension relatively small, subtrapezoidal with rounded corners, in lateral view its posterolateral corner extended slightly posterad to form a hook-like structure; outline of posterior subpetiolar face in lateral view broadly and weakly convex	***M. quadridentata* Eguchi**
8	Anterior border of median clypeal projection subtended by a minute parallel groove bearing approximately 6 stout, pale bristle-like hairs directed anterad. Anteroventral subpetiolar extension relatively large and subrectangular, posteriorly inclined; posteroventral subpetiolar angle obtuse (Indonesia, Bali; gyne only)	***M. balinensis* Taylor & Alpert**
–	Anterior border of anteromedian clypeal projection a single shallowly concave edge, without accompanying groove or hairline. Anteroventral subpetiolar extension small, roughly right-angled-subtriangular; posteroventral subpetiolar angle vestigial and almost absent (Indonesia, Lombok; gyne only)	***M. wallaceana* Taylor & Alpert**

## Supplementary Material

XML Treatment for Metapone murphyi
